# Performance of cohort-adapted dietary and lifestyle inflammation scores among Hispanic adults

**DOI:** 10.3389/fnut.2025.1675057

**Published:** 2026-01-08

**Authors:** Lisa C. Merrill, Sabrina E. Noel, Rafael López Martínez, Josiemer Mattei, Natalia Palacios, Yan Wang, Katherine L. Tucker, Kelsey M. Mangano

**Affiliations:** 1 Department of Biomedical and Nutritional Sciences, University of Massachusetts Lowell, Lowell, MA, United States; 2 The Center for Population Health, University of Massachusetts Lowell, Lowell, MA, United States; 3 Department of Public Health, University of Massachusetts Lowell, Lowell, MA, United States; 4 Department of Computer Sciences, National Autonomous University of Mexico, Mexico City, Mexico; 5 Department of Nutrition, Harvard T.H. Chan School of Public Health, Boston, MA, United States; 6 Department of Psychology, University of Massachusetts Lowell, Lowell, MA, United States

**Keywords:** chronic disease, diet quality, inflammatory cytokines, lifestyle, low-grade inflammation

## Abstract

**Background:**

Dietary and lifestyle inflammation scores (DIS/LIS) were created to assess their contributions to systemic inflammation; however, there is little understanding of their validity in Hispanic adults.

**Objective:**

This study aims to utilize DIS and LIS in the Boston Puerto Rican Health Study (BPRHS), as previously published, and create cohort-adapted scores. Adapted scores were validated in The Puerto Rico Observational Study of Psychosocial, Environmental, and Chronic disease Trends (PROSPECT).

**Methods:**

This cross-sectional analysis assessed diet from a food-frequency questionnaire, self-reported lifestyle information, and inflammation from fasted blood. Published food and lifestyle groups were used to create DIS-1 and LIS-1. Cohort-adapted food and lifestyle groups were used to create DIS-2 and LIS-2 in the BPRHS (*n* = 854). Factor analysis was used to create DIS-3. The associations among DIS-1, LIS-1, DIS-3, and a biomarker score (continuous and dichotomized) were tested in BPRHS using multivariable linear and logistic regression. The associations among DIS-2, LIS-2, DIS-3, and inflammation (continuous concentration and higher versus lower hsCRP) were tested in PROSPECT (*n* = 835) using multivariable linear and logistic regression.

**Results:**

In BPRHS, DIS-1 showed a 1.07 (95% CI: 1.01, 1.15) times greater odds of a high inflammation BMS with each 1-unit increase in the DIS-1 and a 2.86 (95% CI: 2.14, 3.56) times greater odds of a high BMS with each 1-unit increase in the LIS-1. In PROSPECT, LIS-1 showed a 2.91 (95% CI: 2.33, 3.67) times greater odds of high hsCRP with each 1-unit increase in LIS-1; results were similar in linear analyses. DIS-3 was characterized by three factors. In BPRHS, high (quintile 5) vs. low (quintile 1) adherence to factor 1 (healthy diet) showed a 0.57 (95% CI: 0.35, 0.92) times lower odds of high BMS. DIS-3 was not associated with high hsCRP in PROSPECT.

**Conclusion:**

Predetermined lifestyle inflammation scores were associated with inflammation in this population, but dietary inflammation scores were inconsistent in their association with inflammation. Cohort-specific adaptation did not improve the scores’ association with inflammatory status. Further work is needed to understand the role of diet in the development of inflammation in these populations of Hispanic adults.

## Introduction

Low-grade inflammation, defined as elevated concentrations of inflammatory markers in systemic circulation for a period of weeks to years, underlies many of the chronic diseases of aging. This mechanism is bidirectional, such that chronic diseases also contribute to inflammation, driving further disease (1–3). More than 50% of the U.S. population has at least one major chronic disease, 42% have multiple chronic diseases, and these numbers are projected to increase (4–8). In addition to the negative impact these diseases have on quality of life, the management and treatment of chronic diseases exact a financial toll, representing the largest portion of the nation’s annual $4.5 trillion healthcare expenditure (9). Diet and lifestyle are recognized as modifiable risk factors for age-related chronic diseases and may be linked to an individual’s inflammatory status (10–14).

Diet and lifestyle may cause the initial insult to induce the inflammatory response; however, the mechanisms of action remain unclear (15, 16). It is hypothesized that tissue damage results from oxidative stress related to excessive alcohol intake, tobacco use, consumption of energy-dense foods, pesticides, food additives, and excess energy intake (13). Once a chronic disease develops, the continued release of pro-inflammatory molecules such as adipokines, cytokines, and prostaglandins leads to a self-perpetuating cycle of oxidative damage and inflammation that sets the stage for the development of additional disease (13, 17).

The burden of chronic diseases and inflammation has been shown to differ by race and ethnicity (18–21). The prevalence of chronic disease is higher in Puerto Rican (PR) adults, compared to other racial and ethnic groups (22–24), and high serum CRP has been observed (25, 26). Measures directed at reducing systemic inflammation and the subsequent development of chronic diseases in PR adults are urgently needed. Dietary scores aimed to estimate inflammatory burden are one way to identify influencers of inflammation. A more recent inflammatory index score, created by Byrd et al. (27), differs from other dietary inflammation scores in that it provides a strong focus on foods consumed, not on individual nutrients, nor on spices not commonly consumed in some populations. In addition, it is the first index to assess the collective contributions of lifestyle characteristics, including diet, to inflammation. The development and validation of the dietary inflammation score (DIS) and lifestyle inflammation score (LIS) were carried out in populations of adults who identified as non-Hispanic white (NHW) or non-Hispanic black; their validity in a population of Hispanic adults is unknown. The culturally distinct dietary patterns and lifestyle behaviors of Hispanic populations differ from those of others and include high intake of fruit juices and diet beverages (e.g., diet soda), consumption of fruits and starchy vegetables native to the Caribbean, healthy oil intake from vegetables such as avocado, high consumption of homemade fried foods, sedentary behavior, and high prevalence of obesity. Therefore, the objectives of the current study were to 1) apply the Byrd dietary and lifestyle inflammation scores, as published (27) to U.S. mainland PR adults, 2) create cohort-adapted dietary and lifestyle components and score weights, 3) test the newly created components and weights in native island Hispanic adults, and 4) perform factor analysis on the cohort-adapted food components.

## Methods

### Study population (development cohort)

**Figure 1:**
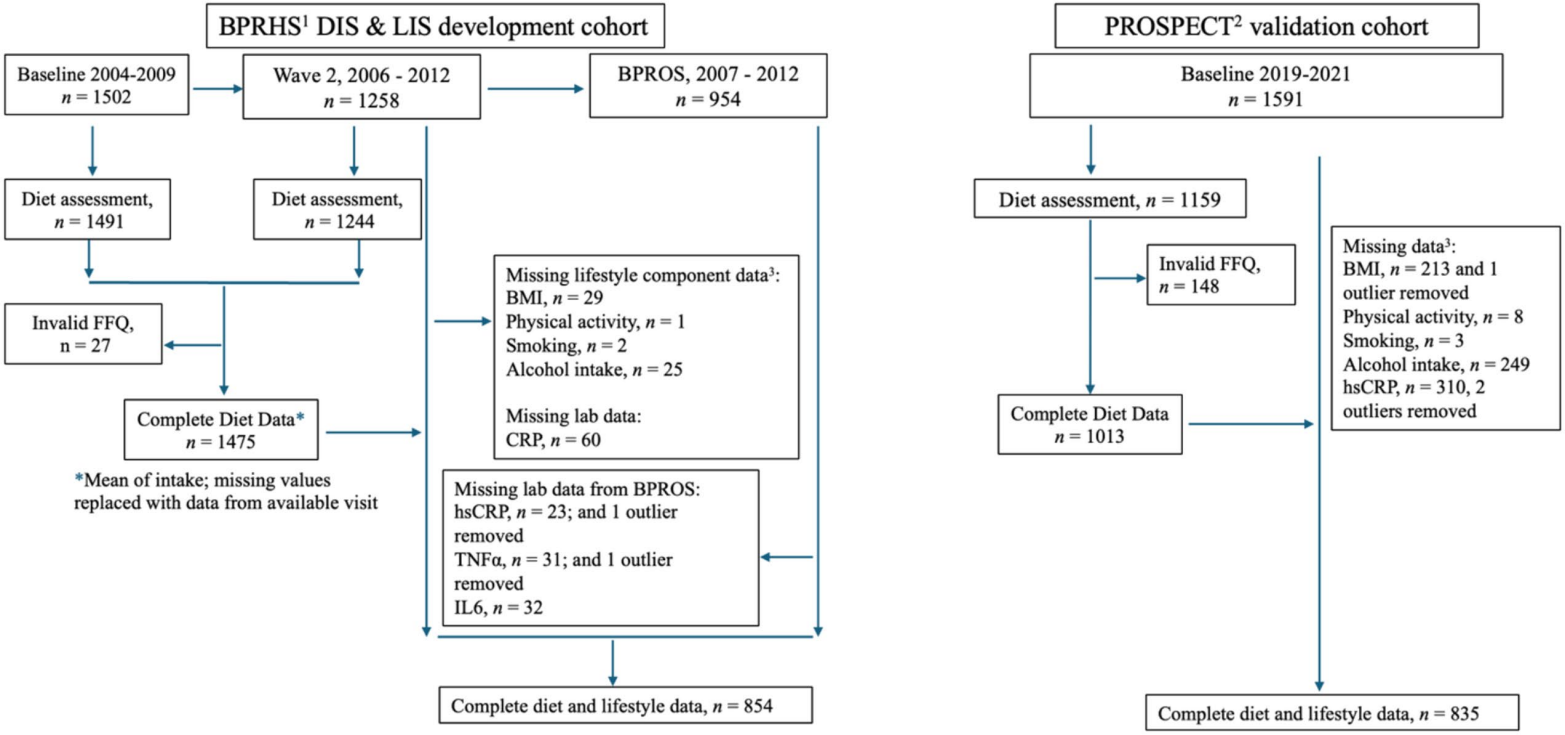
Flowchart of the BPRHS cohort (development cohort) and the PROSPECT cohort (validation cohort) included in the analyses. ^1^BPRHS, Boston Puerto Rican Health Study. ^2^PROSPECT, The Puerto Rico Observational Study of Psychosocial, Environmental, and Chronic disease Trends. ^3^Some participants are missing data from multiple lifestyle score components.

The current cross-sectional analysis included eligible participants from the Boston Puerto Rican Health Study (BPRHS) (Figure 1) to determine whether the dietary inflammation score (mean intake from years 2004–2012) and the lifestyle inflammation score (2006–2012) predict systemic inflammation (2007–2012). The BPRHS is a longitudinal population study investigating numerous chronic health conditions among U.S. mainland PR adults, aged 45–75 years, living in the greater Boston, MA area. From 2004 to 2009, initial baseline interviews were completed by participants (*n* = 1,502), providing information on sociodemographic characteristics, health and health behaviors, dietary intake, migration history, medication use, and stress. Follow-up interviews with 1,258 participants occurred at wave 2 (2006–2012). Participants who completed wave 2 were invited to participate in the ancillary Boston Puerto Rican Osteoporosis Study (BPROS, 2007–2012). Of those invited, 954 completed the interviews and provided biological measurements. All interviews were conducted in participants’ homes by trained, bilingual interviewers. Anthropometric and biological samples (12-h urine and fasted blood at all visits) were also collected. This study was approved by the Institutional Research Board of the University of Massachusetts Lowell.

Dietary intake was assessed at baseline and wave 2 using a semi-quantitative food frequency questionnaire (FFQ) designed and validated for use in this population (28–30). Nutrient intake was calculated using Nutrition Data System for Research software version 2007 (Nutrition Coordinating Center, University of Minnesota) and determined as the mean of foods from baseline and wave 2. If a participant had dietary data from only one time point, that value was used as an estimate of mean intake (*n* = 326). Twenty-seven participants were excluded for invalid FFQ (energy intake of <600 or > 4800 kcal/d) (Figure 1), resulting in 1,475 participants with complete dietary data. All dietary data preceded inflammatory outcome measurements.

Self-reported frequency of alcohol intake (drinks per day) in the past 12 months, current smoking status (any or no smoking), frequency of physical activity, hours of sleep, and perceived stress were assessed as part of a health behavior questionnaire (details outlined below). Trained study personnel obtained measures of height using a SECA 214 portable stadiometer and weight using a Toledo Weight Plate, model I5S clinical scale (Bay State and Systems, Inc.). These were used to calculate body mass index (BMI, kg/m^2^). BMI values >70 kg/m^2^ were determined to be implausible and removed from the analysis (1 in the validation cohort). The physical activity index was assessed as a modification of the Paffenbarger questionnaire, representing the sum of usual daily activity, including hours spent sleeping, and performing sedentary, light, moderate, or heavy activities weighted for the oxygen consumed during each activity (22). Stress was assessed from the Perceived Stress Scale-14, a measure of feelings and thoughts of stress in the past month.

Systemic inflammation was assessed using multiple blood markers [hsCRP, tumor necrosis factor-alpha (TNFα), and interleukin-6 (IL-6)] and represented by a standardized biomarker score (BMS). In brief, the BMS was created by summing standardized scores across all available inflammation biomarkers. hsCRP, IL-6, and TNFα were natural log-transformed, *z*-score standardized, and summed (27), with a higher BMS value representing higher systemic inflammation. Other markers of stress and illness were also measured and controlled in analyses. WBC count and glucose were measured from blood, and cortisol from urine in samples collected at the wave 2 visit of the BPRHS. See Supplementary methods for full details regarding laboratory procedures.

### Study population and data collection (validation cohort)

Cross-sectional analyses were also conducted with eligible participants from the Puerto Rico Observational Study of Psychosocial, Environmental, and Chronic disease Trends (PROSPECT) (Figure 1) to determine whether the dietary and lifestyle inflammation scores, as published and cohort-adapted, were associated with hsCRP (2019–2021) in this cohort. PROSPECT is a prospective cohort study designed to investigate the risk factors for cardiovascular disease among adults aged 30–75 years living in Puerto Rico. Baseline interviews were completed by participants (*n* = 1,591), including data on dietary intake, socioeconomic and environmental factors, medical history, health conditions, lifestyle behaviors, and psychosocial status. The sampling strategy for PROSPECT was designed to provide a representative sample of adults from across the island and is largely comprised of native Puerto Rican individuals. This study was approved by the institutional review boards of the Harvard T.H. Chan School of Public Health, Ponce Health Sciences University (Ponce, PR), and the University of Massachusetts Lowell.

A total of 148 participants were excluded for invalid FFQ (energy intake of <600 or >4,800 kcal/d) (Figure 1), resulting in 1,011 participants with complete dietary data. Fasted blood samples were collected by a licensed nurse; anthropometric measurements and all interviews were conducted in partner clinics by trained research assistants (31). Measurement of serum hsCRP was conducted at the Toledo Clinical Laboratory. See the Supplementary methods for further details.

### Dietary inflammation score components (DIS-1) from published components and weights

Foods, micronutrients, and beverages were grouped into 19 components, closely following the published methods by Byrd et al. (27), to create the DIS-1. Grams of food intake were collapsed into food groups, and mixed dishes were disaggregated and assigned to the appropriate food groups. Food group intakes were assigned to the appropriate DIS component in servings/d, summed, and *z*-score standardized by sex. Components were multiplied by published component weights and summed to obtain a DIS-1 score per participant (27). The same method was followed in the PROSPECT cohort, except that only one visit of dietary data was available for use in creating the score.

Dietary supplement use was also included in the Byrd DIS-1. Due to minimal intake of supplements in the BPRHS and the homogeneity of intake between men and women, an alternative method of ranking intake was employed. Additionally, intake was not grouped by sex. Clinically relevant cut points were used for each supplement included in the score based on Office of Dietary Supplements guidelines,^1^ with 0, 1, or 2 assigned to the lowest, middle, and highest level. Each level was multiplied by +1 for anti-inflammatory supplements (vitamins A, B-12, B-6, C, D, and E, thiamin, niacin, riboflavin, folate, zinc, and calcium) and by −1 for the pro-inflammatory supplement (iron) (32). These values were summed for each participant and included in the DIS-1.

### Lifestyle inflammation score components (LIS-1) from published components and weights

Aspects of lifestyle were grouped into four categorical components following published methods (27), including frequency of alcohol intake, none, moderate: 1 drink per day in women or 1–2 drinks per day men, or heavy: > moderate within the past year; BMI (*<* 25 kg/m^2^, 25–29.9 kg/m^2^, ≥ 30 kg/m^2^); physical activity, sedentary (no meaningful activity) or active (any activity, including light to moderate); and smoking (current smoker vs. not). Activity was limited to two categories instead of three due to the absence of heavy activity. Published weights were multiplied by each level, with the published weight for moderate physical activity applied to the active category in the current study. Values were summed to obtain a LIS-1 score per participant. As with the DIS, the same method was followed in the PROSPECT cohort.

### Development of a cohort-adapted DIS and LIS (DIS-2 and LIS-2, respectively)

Cohort-specific adaptations of the DIS-1 and LIS-1 were performed in the BPRHS. Foods, micronutrients, and beverages were categorized into anti-inflammatory and pro-inflammatory components, starting with the published components but modified to reflect both the unique dietary intake of the BPRHS cohort as well as additional food groups hypothesized to be important contributors to inflammation in this population (27). New food groups developed specifically for the DIS-2 included as proinflammatory were processed dairy, fast foods, fried foods, non-saturated oils (excluding olive oil due to the low quality of the oil consumed in this cohort), condiments, and diet beverages and as anti-inflammatory eggs, fruit juices, starchy vegetables, nuts & seeds, and whole grains (Supplementary Table 1). To avoid multicollinearity, food groups with Pearson’s correlation coefficients >0.3 were combined based on similarity in dietary consumption The following dietary components were combined into a single processed foods component: all fruits and vegetables, fish and starchy vegetables, added sugars with coffee and tea, low-fat dairy and whole grains, processed meats, processed dairy, other fats, refined grains, and fast and fried foods. Sixteen dietary components were included in the final DIS-2 (Table 1) with VIF values from 1.03 to 1.34, reflecting negligible to minimal multicollinearity (33, 34).

**Table 1:** Final derived dietary inflammatory score and lifestyle inflammatory score component weights using data from the Boston Puerto Rican Health Study (2004–2012).

Component group	Foods included	Weights^1^
DIS components^2^		
Fruits and vegetables	Fresh apples, pears, strawberries, blueberries, raspberries, cherries, stewed unsweetened apples, bananas, fresh pasteles, kiwi, grapes, grapefruit, honeydew melon, carrots, apricot fresh and dried, fresh cantaloupe, nectarine, papaya, peach, spinach, lettuce (all types), mustard greens, Brussels sprouts, cabbage, cauliflower, broccoli, parsley, watercress, tomatoes, tomato juice, tomato sauce, salsa, Hispanic salsa verde, asparagus, beets, garlic, peppers, okra, mushrooms, onion, green beans	0.04
100% fruit juice	100% fruit juices, lemon and lime fresh juice, apple juice or cider	0.04
Legumes	Black beans, pink beans, cow peas, pigeon peas, pinto beans, lentils, other beans (excluding soybeans)	0.06
Fish, seafood, and starchy vegetables	Bacalao, cod, haddock, salmon, scallops, sardines, tuna fish, crayfish, shellfish, cassava, plantains (boiled or baked), corn, green peas, potatoes, winter squash, tannier, turnip, pasteles	−0.01
Poultry	Chicken or turkey (with and without the skin, light and dark meat), ground turkey	−0.09
Red and organ meats	Hamburger, beef, lamb, beef liver, chitterlings, kidney, or tongue	0.13
Processed foods	Bacon, beef or pork hotdogs, sausage, other processed meats, American cheese (processed, slices, and spread), Velveeta, coffee creamer (liquid and powder), dips, mayonnaise, margarine, butter, vegetable oil, olive oil, fried rice, fish, beef, clams, crab, scallops, shrimp, crayfish, chicken, plantains, eggrolls, doughnuts, hashed browns, fast food hamburgers, chicken sandwiches, fried chicken, fish fillet sandwiches, French fries, onion rings, breakfast sandwiches, pizza, turnovers, condensed can soup, non-whole grain cold and cooked cereals and breads, bagels, English muffins, rolls, corn bread, white rice and pasta, pancakes, waffles, crackers, pretzels, cookies, cakes, brownies, doughnuts, pie, sweet rolls, coffee cake, dumplings, pudding, custard, pop tarts, granola/protein bars	0.11
Added sugars, coffee, and tea	Sugar-sweetened soda, fruit drinks, nectar, iced tea (sweetened), Gatorade, lemonade, hot chocolate, sweetened coconut meat, chocolate candy bars, other mixed candy, jams, jellies, preserves, syrup or honey, dried or canned fruit, catsup, barbecue sauce, sweet and sour sauce, sweet pickle relish, cranberry sauce, popsicles, sorbet, coffee (decaffeinated and regular), non-herbal and herbal tea	−0.07
High-fat dairy	Whole milk, 2% milk, cream, high-fat ice cream, high-fat yogurt, cream cheese, other high-fat cheeses	−0.15
Low-fat dairy and whole grains^3^	1% milk, skim milk, low-fat yogurt, low-fat ice cream, low-fat cottage cheese or ricotta cheese, low-fat cheeses, whole grain cold cereals, oatmeal, hot-air popcorn, brown rice, whole wheat tortilla, whole wheat bread	0.10
Nuts and seeds	Peanut butter, nuts, sunflower seeds	−0.12
Non-saturated oil	Avocado, guacamole, green and black olives	0.11
Eggs	Eggs: scrambled, fried, boiled, salad	0.15
Condiments	High salt: gravy (canned), horseradish, mustard, dill pickles, soy sauce	0.09
Diet beverages	Diet soda, diet bottled or powdered iced tea, misc. diet drinks (Slim Fast)	0.0
Supplement score^4^	Vitamins A, B-12, B-6, C, D, E; folate, niacin, riboflavin, calcium, magnesium, selenium, thiamin, zinc, β carotene, iron	−0.03
LIS components^5^		
Heavy drinker	>1 drink for women; >2 drinks for men versus none	−0.31
Moderate drinker	1 drink for women; 1–2 drinks for men versus none	−0.52
Physically active	Any activity versus sedentary	−0.66
Current smoker	Current smoker versus does not currently smoke	0.37
Too little sleep	5–6 h of sleep versus 7–8 h	−0.01
Too much sleep	9–10 + h of sleep versus 7–8 h	0.0
High stress	High perceived stress versus low	0.06

^1^Weights are estimates obtained from multivariable linear regression models performed in the BPRHS cohort, representing the average change in an inflammation biomarker score (a summed score comprised of logged and *z*-score standardized hsCRP, IL-6, TNFα) per 1 SD increase in a dietary component or the presence of a lifestyle component. A positive estimate suggests that the component has a proinflammatory effect, while a negative estimate suggests an anti-inflammatory effect. The final regression model was adjusted for age, sex and estrogen status, white blood cell count, urinary cortisol, and all components of the DIS and LIS. ^2^Mean servings per day from baseline and wave 2 dietary intake were *z*-score standardized by sex. ^3^Defined as whole grain first ingredient; added sugar content was ≤ 12 g/serving and DFIB was ≥3 g/serving. ^4^Supplement intakes were based on multivitamin and mineral use. Individuals were ranked into groups based on the office of dietary supplements (ODS) recommended intake of each micronutrient. ^5^Lifestyle components were coded as categorical variables with “0” representing the referent category and “1” and/or “2” the non-referent category.

The LIS was adapted to focus on lifestyle choices; therefore, BMI was excluded, and perceived stress and quantity of sleep (7–8 h recommended, vs. less or more) were included based on recent literature suggesting their association with inflammation (35–38). Perception of stress was defined as high stress (no, yes) based on the PSS-14 scale, dichotomized at the median of 24. Frequency of alcohol intake, current smoker, and physical activity were categorized as described above. Weights from the final model were applied (Table 1).

Unique weights (*β*-coefficients) for each score component within the DIS-2 and LIS-2 were estimated in a single multivariable linear regression model with the inflammation BMS as the dependent variable. Model covariates included for testing were based on published methods and the literature; they were retained if associated with a 10% or greater change in the beta coefficient of ≥40% of the DIS and LIS component predictors (27, 39–41). This resulted in a final model with all components of the dietary and lifestyle scores plus the following relevant covariates: age (y), a single variable combining sex and estradiol status [three levels: men, women with estradiol (exogenous or endogenous), and women without estradiol (post-menopausal, not taking exogenous estradiol), WBC count (1,000/mL), and urinary cortisol (mg)]. The food and lifestyle components were multiplied by their respective weights and summed to obtain final dietary inflammation and lifestyle inflammation scores for each participant (Table 2). While BMI was not included as a lifestyle component in the adapted score (as it is not a lifestyle choice, but reflective of a chronic health condition), we do recognize that BMI is a reflection of lifestyle choices and has a bidirectional association with systemic inflammation; therefore, the DIS-2 and LIS-2 were also created including BMI as a potential covariate in the regression model to obtain cohort-adapted component weights that could be contrasted to the DIS-2 and LIS-2 model without BMI (Supplementary Table 2) (42–44).

**Table 2:** Descriptive characteristics of participants from wave 2 of the BPRHS (2006–2012) and from PROSPECT (2019–2021) with dietary and lifestyle data, and inflammatory cytokine measures.

Characteristics	BPRHS participants mean ± SD or % (*n* = 854)	PROSPECT participants mean ± SD or % (*n* = 835)
Score range		
Dietary inflammation score^1^, as published	−11.69 to 8.06 −0.03 ± 2.4	−10.99 to 6.18 −0.05 ± 2.4
Lifestyle inflammation score^2^, as published	−0.84 to 2.37 1.0 ± 0.6	−0.84 to 2.37 0.77 ± 0.7
Dietary inflammation score, adapted	NA	−1.37 to 1.48 0.0 ± 0.3
Lifestyle inflammation score, adapted	NA	−1.24 to 0.43 −0.41 ± 0.4
Demographics		
Age, y	58.4 ± 7.3	53.3 ± 11.4
Females, %	73.3	75.4
More than high school education, %	16	77.9
Medical history, %		
Has comorbidity^3^	39.7	27.0
HRT user	0.01	7.7
Lifestyle, %		
Current smoker	20.7	9.9
Normal BMI	10.5	18.6
Nondrinker	65.9	49.3
Sedentary	79.5	67.9
Low perceived stress	53.9	52.4
Adequate sleep (7–8 h)	40.7	55.3
Dietary intakes		
Total energy, kcal/d	2029 ± 753	2,694 ± 944
Carbohydrates, % kcal/d	55.1 ± 12	43.4 ± 8.5
Dietary fiber, g/(1,000 kcal/d)	10.7 ± 3.5	8.8 ± 2.6
Total fat, % kcal/d	34.1 ± 8.7	39.5 ± 6.1
Polyunsaturated fat, g/(1,000 kcal/d)	10.0 ± 3.0	9.7 ± 2.0
Monounsaturated fat g/(1,000 kcal/d)	13.1 ± 3.5	15.9 ± 3.0
Protein, % kcal/d	18.4 ± 4.6	17.4 ± 3.0
Urinary cortisol (mg)	38.8 ± 32.8	NA
White blood cell count (1,000/uL)	6.8 ± 2.1	NA
Inflammatory markers		
Plasma hsCRP >3 mg/L^4^, %	57.9	48.0
Plasma hsCRP (mg/L)	6.5 ± 9.7	5.5 ± 7.7
Plasma IL-6 (pg/mL)	4.0 ± 4.2	NA
TNF-α (pg/mL)	2.4 ± 2.7	NA

^1^Combined baseline and wave 2 dietary data in the BPRHS. ^2^Wave 2 data in the BPRHS. ^3^Includes self-reported heart disease, cancer (not including skin cancer), and diabetes. ^4^Combined wave 2 BPRHS and baseline BPROS data.

### Validation of cohort-adapted DIS-2 and LIS-2

Validity of the cohort-adapted DIS-2 and LIS-2 was tested in the PROSPECT cohort. Grams of food intake were collapsed into the cohort-adapted food groups, and mixed dishes were disaggregated and assigned to the appropriate food groups in servings/d. Servings were summed and *z*-score was standardized by sex and multiplied by the cohort-adapted weights (Table 1). Lifestyle components were created as above and multiplied by the cohort-adapted weights (Table 1). The food and lifestyle components were summed to obtain a cohort-adapted dietary and lifestyle inflammation score for each participant.

### Exploratory and confirmatory factor analysis of dietary intake, DIS-3

DIS-3 dietary patterns were derived using exploratory factor analysis (FA) in the BPRHS from 26 of the original 29 DIS-2 dietary components (diet beverages, supplements, and coffee and tea components were not included) using the psych package in R (45), rotating the factors orthogonally (varimax option) and specifying the factor method as ordinary least squares. Loadings of factors with eigenvalues >1 were evaluated, including examination of scree plots. To determine the ideal factor solution, a formal comparison was performed. Models were built for a pre-determined number of factor solutions (3–6) over 20 replicates of a random subsampling of the data (sample sizes = 400, 600, 800, and 1,000 individuals). To determine consistency among the first three factors of each model, a distance matrix was built between the vectors of loadings corresponding to the first three respective factors obtained for each of the 20 replicates (e.g., for 3 factors and a subsampling size of 400, we ran the model 20 times and obtained 20 vectors of loadings corresponding to factor 1; a distance matrix was built over these 20 vectors of loadings). The mean and SD of these distances were calculated, as was the general average for both quantities over all sample sizes. The hypothesis was that the lowest values represented the most consistent final model; based on this hypothesis, a 3-factor solution was retained (Supplementary Table 3). For each factor, a score for each participant was calculated by summing intakes of food groups weighted by their factor loadings (≥ |0.20|). The validity of the exploratory FA factor structure was tested using confirmatory factor analysis (CFA) in the PROSPECT cohort. Confirmatory dietary patterns were derived using the CFA function in the lavaan package in R (46). The MLM (maximum likelihood estimation) estimator was specified due to the non-normal distribution of the data. MLM is a maximum likelihood estimation method with robust standard errors and a Satorra–Bentler scaled test statistic. This method improves the accuracy of the standard errors and indexes of model fit (47, 48). Food groups from the exploratory FA that were not represented in the CFA results were removed from the list of food groups, and the exploratory FA was repeated. The newly created exploratory FA factor structure was re-tested via CFA in PROSPECT (including and excluding non-significant foods in each factor); the best model fit was determined based on Akaike Information Criterion (AIC), comparative fit index, root mean square error of approximation, and standardized root mean square residual. The best model included 26 food groups and all factor loadings (including those that were not significant) (Supplementary Table 4). Food groups with a non-zero factor loading in at least one of the three factors were entered into a final CFA model in PROSPECT (fruit juice, starchy vegetables, non-saturated oil, and condiments were excluded). For each factor, a score for each participant was calculated by summing intakes of food groups weighted by their respective factor loadings.

### Statistical analysis

Means ± SD for continuous variables and proportions of participants for categorical variables were calculated for the study populations. Normality was determined by visual inspection of histograms and linearity between predictor scores and outcome variables by inspection of scatterplots. The inflammation BMS score was used as a continuous dependent variable in models creating weights for DIS-2/LIS-2 in the BPRHS, but as a categorical variable (dichotomized at the median of −0.23 into higher vs. lower inflammation) for models testing associations with the DIS-1/LIS-1 and DIS-3 per the published methods. hsCRP was also dichotomized into high vs. lower inflammation, defined by clinical standards at 3.0 mg/L (27) for tests of association with DIS-1/LIS-1, DIS-2/LIS-2, and DIS-3 in PROSPECT.

Multivariable logistic regression was used to associate DIS-1/LIS-1, DIS-2/LIS-2, and DIS-3 with markers of inflammation as defined above (higher versus lower). The published and cohort-adapted DIS and LIS weights were created by including a comprehensive list of potential confounders of the primary associations with inflammatory cytokines. Covariates for the logistic regression models were chosen based on previous literature and retained if they caused a 10% or greater change in the beta coefficient of the predictor (39). In the BPRHS, the DIS-1 and LIS-1, calculated using published components and weights, were associated with the inflammation BMS. The DIS-1 model was adjusted for age and a single variable combining sex and estradiol status. The LIS-1 model was adjusted for age (y) and sex and estradiol status. In PROSPCT, the DIS-1/LIS-1 and DIS-2/LIS-2 were associated with serum hsCRP concentration. The DIS-1 model was adjusted for the following relevant covariates: age, a single variable combining sex and estradiol status, LIS components (BMI, frequency of alcohol intake, current smoker, and physical activity), comorbidities [history of heart disease, cancer, or diabetes (all y/n)], and total energy (kcal/d). The LIS-1 model was adjusted for age and sex and estradiol status. The DIS-2 model (without BMI as a covariate in the weight-building model) was adjusted for age, sex and estradiol status, comorbidities, and total energy. The LIS-2 model (without BMI as a covariate in the weight-building model) was adjusted for age and sex and estradiol status. Odds ratios (OR) and 95% CI were calculated. The DIS-2 model (with BMI as a covariate in the weight-building model) was adjusted for age, sex and estradiol status, lifestyle components, comorbidities, total energy, and education. The LIS-2 model (with BMI as a covariate in the weight-building model) was adjusted for age, sex and estradiol status, comorbidities, and education. Odds ratios (OR) and 95% CI were calculated. Per published methods, LIS components were tested in regression models together, as were comorbidities. A sensitivity analysis was conducted testing associations between the DIS-1/LIS-1, DIS-2/LIS-2, and DIS-3 and markers of inflammation, modeled as continuous variables. hsCRP in PROSPECT was log transformed before inclusion in multivariable linear regression models, which were adjusted for the same covariates as their respective logistic regression models.

Individuals were placed into quintiles based on their exploratory factor analysis (EFA) and CFA factor loadings in BPRHS and PROSPECT, respectively. For each factor, the difference between the relations of the quintiles (Q1 served as the reference group) and inflammation (higher versus lower) was examined in both cohorts using multivariable logistic regression (49), and odds ratios (OR) and 95% CI were calculated. Model adjustment was the same as described for DIS-1 and DIS-2, except lifestyle score components and comorbidities were tested as individual variables. The DIS-3 model in BPRHS was adjusted for the following relevant covariates: age (y), a single variable combining sex and estradiol status, BMI (kg/m^2^), current smoker (y/n), physically active (y/n), history of heart disease (y/n), and history of diabetes (y/n). The DIS-3 model in PROSPECT was adjusted for age (y), a single variable combining sex and estradiol status, BMI (kg/m^2^), current smoker (y/n), perception of stress (y/n), and history of diabetes (y/n). To visualize the EFA and CFA results, dandelion plots were created using the DandEFA and RColorBrewer packages in R (50, 51). All analyses were performed using R Statistical Software (v4.3.0) (52) and included the Tidyverse and sqldf packages (53, 54).

## Results

### Sample characteristics

Descriptive characteristics of participants from the development and validation cohorts (BPRHS and PROSPECT, respectively) are provided in Table 2. Both samples had more female than male adults and were similar in age. Few BPRHS participants had attained more than a high school level of education, compared to ~75% of the PROSPECT participants. More than half of the BPRHS sample and just under half of the PROSPECT sample had serum hsCRP concentration >3 mg/L. Dietary intakes of protein (% kcal/d), fiber, polyunsaturated fatty acids, and monounsaturated fatty acids (each g/1,000 kcal/d) were similar across samples. Compared with PROSPECT, those in the BPRHS reported an average of 664 fewer kcal/d, with 7% less of their total kcal intake from fat, but had higher carbohydrate intake. The BPRHS sample reported more comorbidities, had twice the number of current smokers, relatively more who consumed no alcohol, more who reported sedentary behavior, fewer who reported obtaining the recommended hours of sleep, and fewer with normal BMI. Despite these differences, the two samples showed a similar range of DIS-1, using published food components and weights, and the same LIS-1 range, although the mean and standard deviation for the LIS differed between the two samples. The range of the DIS-2 in PROSPECT, following the application of cohort-adapted components and weights, was narrower than that obtained from published components and weights, while the range of the LIS-2 was similar.

### Associations between published inflammation scores and inflammatory cytokines

Associations of the DIS-1 and LIS-1, created from published components and weights with the inflammation BMS in the BPRHS, and with hsCRP in PROSPECT, are shown in Table 3. Each 1-unit increase in the DIS-1 was associated with 1.08 (95% CI: 1.01, 1.15) times higher inflammation BMS than in the BPRHS cohort, but showed no association with hsCRP in PROSPECT. Each 1-unit increase in the LIS-1 showed 2.86 times (95% CI: 2.20, 3.71) higher inflammation BMS in the BPRHS and 2.91 (95% CI: 2.33, 3.67) times higher hsCRP in PROSPECT. Effect estimates from models run with continuous dependent variables did not change direction nor meaningfully change the magnitude of the estimates or their statistical significance (Table 3).

**Table 3:** Associations between dietary and lifestyle inflammation scores, and inflammation biomarker score or high-sensitivity C-reactive protein in the BPRHS (wave 2) and PROSPECT cohorts.

Inflammation scores	Published components and weights applied in BPRHS^1^	Published components and weights applied in PROSPECT^2^	Cohort-adapted components and weights applied in PROSPECT^2^
Inflammation (high/low)			
	Adjusted OR (95% CI)	Adjusted OR (95% CI)	Adjusted OR (95% CI)
DIS^3,4,5^	**1.07 (1.01, 1.15)**	1.02 (0.95, 1.09)	1.36 (0.84, 2.20)
LIS^6,7,8^	**2.86 (2.20, 3.71)**	**2.91 (2.33, 3.67)**	1.30 (0.89, 1.90)
Inflammation biomarker score (−4.8 to 9.9) or hsCRP (mg/L)			
	*β* (95% CI)	*β* (95% CI)	*β* (95% CI)
DIS^9^	0.06 (0.0, 0.12)	0.01 (−0.01, 0.03)	0.12 (−0.68, 0.31)
LIS^9^	**1.09 (0.85, 1. 3)**	**0.42 (0.35, 0.50)**	0.13 (−0.02, 0.28)

^1^Inflammation biomarker score dichotomized at the median ≤/> − 0.23 for OR; continuous score for *β* coefficients from multivariable linear regression. ^2^Serum hsCRP dichotomized at the median ≤/> 3 mg/L for OR; log-transformed and continuous concentration for β coefficients from multivariable linear regression. ^3^Age, sex and estradiol status, and LIS groups. ^4^Age, sex and estradiol status, LIS groups, total energy, history of diabetes, heart disease, and cancer. ^5^Age, sex and estradiol status, total energy, history of diabetes, heart disease, and cancer. ^6,7^Age and sex and estradiol status. ^8^Age, sex and estradiol status, history of diabetes, heart disease, and cancer. ^9^Same covariates used in linear and logistic regression models.

Bold values indicate *p*-value less than 0.05.

### Cohort-adapted score components and weights

The adapted food groups and weights for the cohort, 16-component DIS-2, and the 5-component LIS-2 are shown in Table 1. The lifestyle components were generally in the hypothesized direction; current smoker status and high perceived stress were positively associated, and being physically active was inversely associated with the inflammation BMS. Higher alcohol intake was less protective against higher inflammation BMS than moderate intake. Relevant beta coefficients were similar to the published LIS weights. The weights of many of the dietary components (Table 1) match the directionality shown by Byrd and colleagues in the REGARDS cohort. However, several cohort-adapted dietary components in the current study showed a relation with the BMS that was in the opposite direction to that hypothesized. For example, the fruits and vegetables and legume components were positively related (classified as proinflammatory), while the added sugars, coffee, and tea components were negatively related (classified as anti-inflammatory). However, numerous beta coefficients were close to zero, suggesting a neutral association with inflammation. Similarly, the beta coefficients of the sleep and perceived stress lifestyle components were zero or close to zero. Both moderate and heavy alcohol use were negatively related to inflammation, in contrast to the published results in which only moderate use showed a negative relation. The range of the lifestyle component weights (LIS-2: −0.66 to 0.37) was wider than that of the dietary component weights (DIS-2: −0.15 to 0.15).

### Associations between cohort-adapted inflammation scores and hsCRP

Neither the DIS-2 nor the LIS-2 was associated with high hsCRP in the PROSPECT cohort (Table 3). Effect estimates from the main models (exclusion of BMI as covariate in the weight-building model) created with hsCRP as a continuous outcome did not change direction; however, the LIS-2 demonstrated a stronger association than was observed in the model created with hsCRP as a dichotomized outcome (estimates: 0.13 and 0.26; *p*-values: 0.09 and 0.18, respectively). Inclusion of BMI as a covariate in the DIS-2 and LIS-2 weight-building model did not improve the performance of the cohort-adapted inflammation scores in PROSPECT (Supplementary Table 4); however, a similar pattern of association between the LIS-2 and hsCRP as a continuous outcome was observed. The effect estimate was attenuated in the continuous model, as above, but the *p*-value was smaller compared with the model created with hsCRP as a dichotomized outcome (estimates: 0.12 and 0.26; *p*-values: 0.04 and 0.18, respectively).

### Associations between empirically derived dietary inflammation scores and inflammatory cytokines

**Figure 2:**
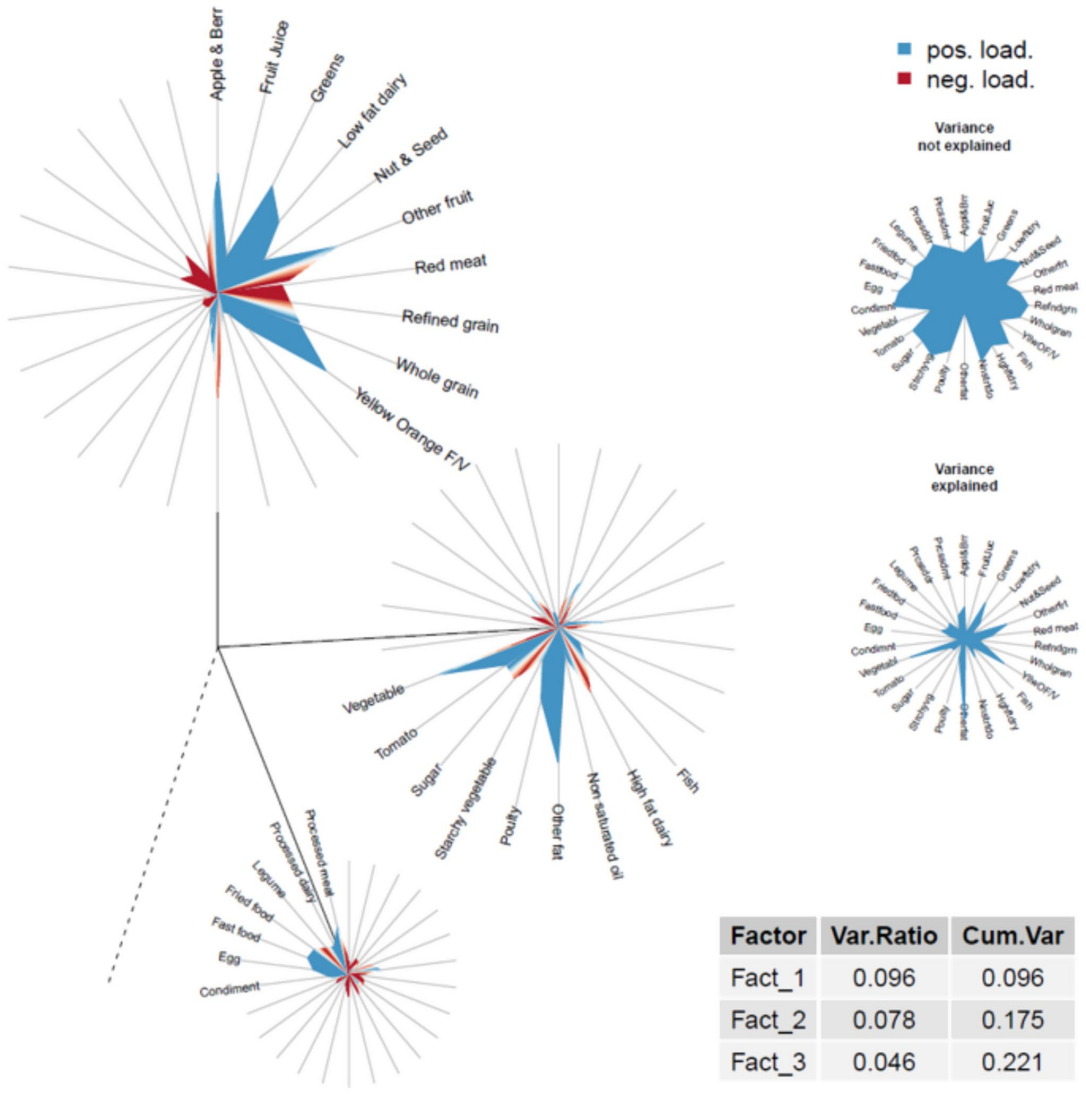
Dandelion plot of three-factor exploratory factor analysis from Boston Puerto Rican Health Study (2004–2012).

**Figure 3:**
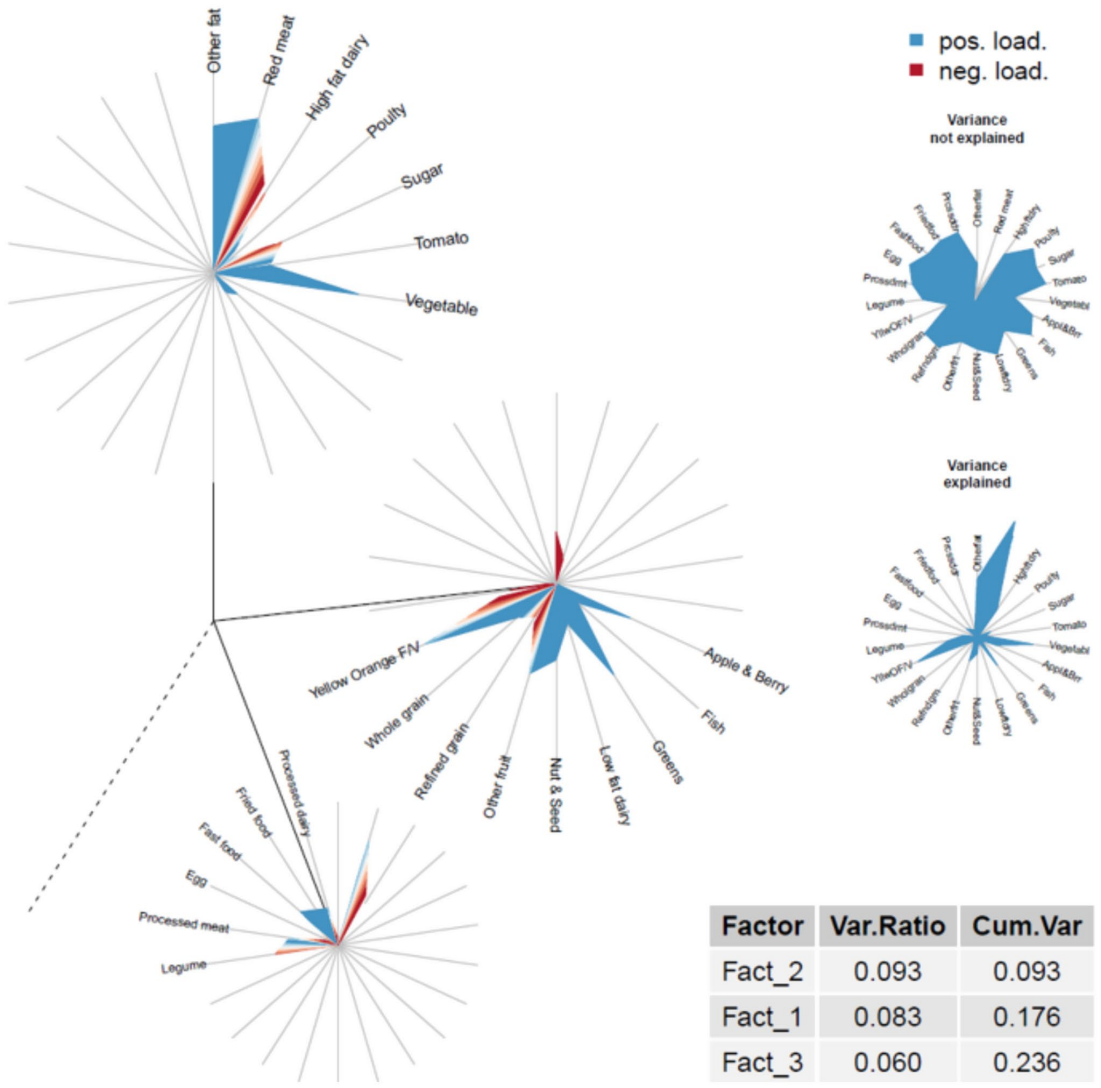
Dandelion plot of three-factor confirmatory factor analysis in The Puerto Rico Observational Study of Psychosocial, Environmental, and Chronic disease Trends (2004–2012).

Three main dietary patterns emerged as most meaningful for this population (Supplementary Tables 5, 6). Factor 1, a healthy pattern, had high factor loadings from fruit and fruit juice, green, yellow, and orange vegetables, low-fat dairy, and whole grains, and low loadings from saturated fat, red and processed meat, and refined grains. Factor 2, a traditional pattern, was characterized by high loadings from saturated fat, beans and legumes, and tomatoes, and low in high-fat dairy and added sugar. Factor 3, an industrialized pattern, had high loadings from red and processed meat, fried foods, and eggs, and low loadings from high-fat dairy, legumes, saturated fat, and fruit. The three factors explained 23.6% of the total variance in PROSPECT and showed a reversal in the order of importance of the traditional and healthy dietary patterns compared with BPRHS (Figures 2, 3). Associations of each DIS-3 quintile derived via exploratory FA in the BPRHS cohort and confirmed in the PROSPECT cohort, respectively, with the inflammation BMS and with hsCRP were performed. In the BPRHS, greater adherence to a healthy dietary pattern (Q5 of factor 1) was associated with a 0.57 (95% CI: 0.35, 0.92) times lower odds of high inflammation, compared to lower adherence to the dietary pattern (Q1). No other quintiles in factors 1, 2, or 3 were associated with high inflammation. No associations with hsCRP were observed in PROSPECT (Supplementary Table 7). Tests for associations between Factors 1–3 and continuous inflammation outcomes revealed a difference in the performance of the association between Q5 of Factor 1 and the inflammation BMS and in the association of Q5 of Factor 2 with hsCRP. While the effect estimates from the continuous and dichotomized outcome models for Q5 of Factor 1 and the inflammation BMS were similar (−0.42 and −0.57, respectively), the strength of the association was attenuated in the continuous model (*p*-values: continuous = 0.07 and dichotomous = 0.03). In PROSPECT, greater adherence to a traditional dietary pattern (Q5 of factor 2) was associated with a 0.24 (95% CI: 0.43, 1.3) decrease in hsCRP, compared with the absence of an association in the dichotomized outcome model.

## Discussion

The Byrd and colleagues’ dietary inflammation score was associated with 1.07 (95% CI: 1.01, 1.15) times higher inflammation, compared to low, in mainland PR adults (BPRHS), but was not associated with inflammation in island-dwelling Hispanic adults (PROSPECT). Cohort adaptation of the dietary inflammation score did not improve its performance in the island-dwelling adults. Greater adherence to a healthy diet, defined by dietary pattern analysis, compared to lower adherence, was associated with lower odds of high inflammation in BPRHS (OR: 0.57; 95% CI: 0.35, 0.92), but not in PROSPECT. Application of the published lifestyle inflammation score was associated with 2.86 (95% CI: 2.20, 3.71) times higher inflammation in BPRHS and 2.91 (95% CI: 2.33, 3.67) times higher inflammation in PROSPECT. Cohort adaptation of the lifestyle inflammation score did not improve its performance in PROSPECT. Overall, predetermined lifestyle inflammation scores were associated with inflammation in these populations, but dietary inflammation scores were inconsistent in their association with inflammation. Additionally, cohort-specific adaptation of the inflammation scores did not improve their association with inflammatory status, although the cohort-adapted lifestyle score was weakly associated with hsCRP as a continuous outcome (*p*-value = 0.09).

Incorporating factors known to influence inflammation into one score provides an opportunity to predict inflammatory status more comprehensively than considering the factors individually. With the increasing prevalence of chronic disease, the use of inflammation scores has the potential to help address an urgent public health need. The creators of the original DIS and LIS signaled their understanding of the importance of creating population-specific scores and made it easy to do so by providing detailed methodological steps in their publication. Following these methods in the current study provided the opportunity to capture the unique aspects of a sample of Hispanic adults and to determine whether the scores were meaningful predictors of systemic inflammation in this population. However, despite cohort-specific adaptations, the adapted DIS did not predict systemic inflammation in the current study. In contrast, the LIS was moderately associated with inflammation. The weaker association with systemic inflammation of the published DIS, in comparison to the published LIS, in both cohorts of Hispanic adults, is consistent with the literature, including the cohorts in which the DIS was created (55–58). Additionally, two studies with healthy adults living in Tehran showed no association of the predetermined DIS with cardiorespiratory fitness (265 healthy adults, aged 18–70 years) or with incident diabetes (4,624 adults, aged 20–75 years) (59, 60), providing further support that considering diet alone in predicting systemic inflammation may not be sufficient.

To complete a comprehensive evaluation of the potential relation of diet with systemic inflammation, beyond the *a priori* assumptions of the DIS, factor analysis was performed to capture dietary patterns of the BPHRS. Three distinct dietary factors were shown, and only the healthy factor was inversely related to inflammatory cytokines, suggesting that diet, particularly in comparison to other lifestyle factors, may not be an important predictor of inflammation in these cohorts of Hispanic adults. In a recent systematic review, Hart and colleagues reported that nearly all empirical dietary patterns and numerous hypothesis-driven patterns failed to predict biomarkers of inflammation (57). Furthermore, among 775 female participants of the Women’s Lifestyle Validation Study (mean age 64 years), three diet quality scores (DASH, aMED, and aHEI) failed to predict adiponectin (58). The numerous neutral weights associated with the DIS components, minimal association between FA dietary patterns and dichotomized and continuous inflammatory cytokines, and the low percent variation in the dataset explained by the factors suggest that alternative factors contribute to the high markers of inflammation in Hispanic adults residing on the island and on the mainland.

It is established that pro-inflammatory cytokines increase with age, although which cytokine(s) increase and the degree to which they increase varies with age, health status, and by race and ethnicity (27). The etiology behind these differences could be the cultural, physiological, and/or socioeconomic contributions to health behaviors unique to each race and ethnicity. The application of scores that do not reflect unique aspects of the represented population may return null or non-significant results and lead to false conclusions. D’Agostino and colleagues described the extensive methodological changes required to successfully apply the Framingham Coronary Heart Disease Prediction Scores in populations that were not non-Hispanic White or Black, specifically among Japanese American and Hispanic men and Native American women (61). Similarly, the use of Polygenic risk scores, based on data from populations of European descent, has been described as contributing to health disparities due to its lack of external generalizability in populations not of European descent (62). Therefore, it appears to be necessary to consider modification of predictive health scores by population. Cultural adaptation of the LIS (LIS-2) attenuated the observed association of the original LIS (LIS-1) with hsCRP in PROSPECT. This may be due to our decision to focus on lifestyle choices, resulting in the exclusion of BMI as a component of the LIS-2, and the inclusion of quantity of sleep and perception of stress. Although not a lifestyle choice, but a reflection of lifestyle choices, BMI can be a part of the development and/or continuation of systemic inflammation. Therefore, it was important to adjust for BMI in the models used to create the LIS. Yet, inclusion of BMI as a covariate in the weight-building regression model did not improve the performance of the LIS-2. These data suggest that BMI, an established marker of health that is associated with inflammation, may have a mediating role between diet, lifestyle, and inflammation (63). To the best of our knowledge, no studies utilizing the DIS/LIS have excluded BMI as a lifestyle component, nor have any included measures of sleep and/or stress, precluding a comparison of our results to others. The weakly positive association between the LIS-2 and the continuous, log-transformed hsCRP outcome raises another potential issue for researchers considering the use of these scores. Of note, while this deviation from the published method approached statistical significance, the effect estimates obtained were attenuated, a phenomenon that was likely due to the loss of power resulting from dichotomization of hsCRP (64). Furthermore, participants in the current study were not excluded based on the hsCRP cut point of 10 mg/L, which was done for the original DIS/LIS, as the current cohort demonstrates higher levels of inflammation compared to other populations (65, 66). Future studies are warranted that capture a wide array of lifestyle choices to further identify lifestyle choices that may influence inflammation, and how they should be captured in a score meant to predict inflammation.

Contrary to our hypothesis, cohort adaptation of the dietary and lifestyle scores (created in a cohort of mainland Puerto Rican adults) did not improve the prediction of inflammation among adults living on the island of Puerto Rico. Despite these two cohorts being of Hispanic descent and from similar cultural backgrounds, they do show different sociodemographic, dietary, and health differences. For example, island-dwelling adults show lower rate of comorbidities, lower hs-CRP levels, lower rates of smoking, and fewer individuals who are sedentary. In addition, there was an observed difference in patterns of overall dietary consumption between the BPRHS and PROSPECT cohorts. Factor analysis suggests greater adherence to a traditional dietary pattern in the island-dwelling Hispanic adults compared to the mainland PR adults. Previous literature documents a high adherence to a neo-traditional dietary pattern in the PROSPECT cohort (56). Greater adherence to the traditional dietary pattern in PROSPECT was associated with lower hsCRP in the continuous outcome model. The importance of differences in patterns of dietary consumption to the performance of the dietary inflammation score is further supported by the observed association in the current study between the published DIS-1, created in a mainland population, and inflammation in mainland PR adults, but the absence of a similar association in the island-dwelling Hispanic adults. While the same FFQ was used in both cohorts, and care was taken to remove invalid reports of dietary intake, potential FFQ measurement error is an additional possible explanation for the weaker association of diet with inflammation in PROSPECT.

Another potentially important explanation for the poor performance of the dietary scores in PROSPECT is the sole use of hsCRP as a marker of inflammation. Solely relying on CRP to represent systemic inflammation is a risk, as it is a downstream marker of inflammation that represents only specific pathways of inflammation and cannot be disentangled from its strong association with adiposity (67). Other studies have also shown null associations of diet with inflammation when only CRP was used. For example, in the EPIC-Potsdam Cohort (68), a study of 636 adults (mean age 50.8 years), adherence to the anti-inflammatory EAT-Lancet and Mediterranean dietary patterns was not associated with plasma hsCRP concentrations. Similarly, a larger study of adults (mean age 49 years) using NHANES data (*n* = 4,760 in 2015–2016 and *n* = 4,982 in 2017–2018) reported no association between adherence to the healthy eating index and hsCRP (69). Furthermore, among 1,352 healthy adults (aged 18–69 years) from the Observation of Cardiovascular Risk Factors in Luxembourg study, the dietary inflammatory index (DII) was not associated with hsCRP concentration (70). Therefore, future studies evaluating the relation of diet with inflammation should consider using a more comprehensive biomarker score to capture all stages of the inflammatory process and may choose not to use hsCRP alone. Finally, DIS component weights were created with lifestyle component groups included in a single linear regression model, and it is possible that any influence of diet was suppressed by lifestyle.

This study is the first to test the validity of the recently published dietary and lifestyle inflammation scores in cohorts of Hispanic adults living in the U.S. mainland and on the island of Puerto Rico. It included repeated measures of dietary intake in the development cohort (BPRHS) via a culturally tailored and validated FFQ. The same FFQ was used in the validation cohort (PROSPECT), and the data were processed in the same way, providing confidence in the accuracy of the self-reported responses. This study also used data from three inflammatory cytokines to develop a biomarker score in the BPRHS cohort. Furthermore, the study included a sensitivity analysis of the associations of the dietary and lifestyle inflammation scores and dietary factors with continuous outcome variables. A limitation of the biomarker score is the absence of an anti-inflammatory cytokine, such as IL-10, which would have provided a countermeasure to the proinflammatory cytokines. With only hsCRP measured in participants of PROSPECT, the authors were unable to test whether the DIS and LIS scores were predictive of alternative inflammatory cytokines. Health behaviors and medical history were based on self-report and, therefore, subject to error. As with all observational studies, the potential for residual confounding exists in this study, but was minimized by careful adjustment for potential confounders. Additionally, this was a cross-sectional study, limiting its ability to determine causality.

These findings have important public-health implications for efforts to reduce inflammation-related chronic disease among Puerto Rican and other Hispanic adults. The limited and inconsistent associations between dietary scores and inflammation observed in this study suggest that diet may not be an impactful contributor to inflammatory risk in these populations. Instead, our results raise the possibility that lifestyle factors such as smoking, alcohol intake, and physical activity may exert a stronger influence on systemic inflammation, although further work is needed to elucidate the role of diet and BMI. Identifying the relative contributions of diet and lifestyle factors to inflammation will be essential for designing meaningful approaches to reduce disparities in chronic disease among Hispanic adults.

## Conclusion

A published dietary inflammation score, created in NHW and NHB cohorts, was weakly associated with an inflammatory cytokine biomarker score in mainland PR adults but was not associated with inflammation in Hispanic adults living on the island (assessed by hsCRP). Cohort adaptation of the diet score did not improve the association with inflammation in a genetically similar group of Hispanic adults living on the island. Greater adherence to a healthy diet, as determined from factor analysis, showed lower odds of high inflammation in mainland PR adults but was not associated with inflammation in Hispanic adults living on the island. Differences between the cohorts in dietary patterns and measures of inflammation are suspected reasons for the inconsistent associations observed. Further work is needed to understand whether there are individual dietary determinants of inflammation in these populations of Hispanic adults, or whether inflammation is driven by overall intake relative to need (BMI as a surrogate measure), to effectively guide future health disparities research.

## Data Availability

The raw data supporting the conclusions of this article will be made available by the authors, without undue reservation.

## Footnotes

1
https://ods.od.nih.gov/factsheets/list-all/#C
