# Supplementary material for: Performance of cohort-adapted dietary and lifestyle inflammation scores among Hispanic adults
Source: Front Nutr. 2026 Jan 8;12:1675057. doi: 10.3389/fnut.2025.1675057 (PMC12823488; doi:10.3389/fnut.2025.1675057)
Supplement: Supplementary file 1 [file Table_1.DOCX]

**Supplementary Table 1.** Original derived Dietary Inflammatory Score and Lifestyle Inflammatory Score component weights using data from the Boston Puerto Rican Health Study (2004-2012).

| Component Group | Foods included | Weights^1^ |
| --- | --- | --- |
|  |  |  |
| DIS-1 components^2^ | | |
| Greens and cruciferous vegetables | Spinach, lettuce (all types), mustard greens, Brussels sprouts, cabbage, cauliflower, broccoli, parsley, watercress | 0.10 |
| Tomatoes | Tomatoes, tomato juice, tomato sauce, salsa, Hispanic salsa verde | -0.06 |
| Apples and berries | Fresh apples, pears, strawberries, blueberries, raspberries, cherries, stewed unsweetened apples | -0.15 |
| Yellow/orange fruits and vegetables | Carrots, apricot fresh and dried, fresh cantaloupe, nectarine, papaya, peach | 0.05 |
| Other fruit | Fruit other than those listed above (e.g., bananas, fresh pasteles, kiwi, grapes, grapefruit, honeydew melon | 0.0 |
| 100% Fruit juice | 100% fruit juices, lemon and lime fresh juice, apple juice or cider | 0.05 |
| Other vegetables | Vegetables other than those listed above (e.g., asparagus, beets, garlic, peppers, okra, mushrooms, onion, green beans) | -0.03 |
| Legumes | Black beans, pink beans, cow peas, pigeon peas, pinto beans, lentils, other beans (excluding soybeans) | 0.09 |
| Fish and seafood | Bacalao, cod, haddock, salmon, scallops, sardines, tuna fish, crayfish, shellfish | -0.04 |
| Poultry | Chicken or turkey (with and without the skin, light and dark meat), ground turkey | -0.11 |
| Red and organ meats | Hamburger, beef, lamb, beef liver, chitterlings, kidney, or tongue | 0.14 |
| Processed meat | Bacon, beef or pork hotdogs, sausage, other processed meats | 0.18 |
| Added sugars | Sugar-sweetened soda, fruit drinks, nectar, iced tea (sweetened), Gatorade, lemonade, hot chocolate, sweetened coconut meat, chocolate candy bars, other mixed candy, jams, jellies, preserves, syrup or honey, dried or canned fruit, catsup, barbecue sauce, sweet and sour sauce, sweet pickle relish, cranberry sauce, popsicles, sorbet | -0.07 |
| High-fat dairy | Whole milk, 2% milk, cream, high-fat ice cream, high-fat yogurt, cream cheese; other high-fat cheeses | -0.12 |
| Low-fat dairy | 1% milk, skim milk, low-fat yogurt, low-fat ice cream, low-fat cottage cheese or ricotta cheese, low-fat cheeses | 0.11 |
| Processed dairy | American cheese (processed, slices, and spread), Velveeta, coffee creamer (liquid and powder), dips | 0.16 |
| Coffee and tea | Coffee (decaffeinated and regular), non-herbal and herbal tea | 0.00 |
| Nuts and seeds | Peanut butter, nuts, sunflower seeds | -0.07 |
| Other fats | Mayonnaise, margarine, butter, vegetable oil, olive oil | 0.07 |
| Refined grains | Non-whole grain cold and cooked cereals and breads, bagels, English muffins, rolls, corn bread, white rice and pasta, pancakes, waffles, crackers, pretzels, cookies, cakes, brownies, doughnuts, pie, sweet rolls, coffee cake, dumplings, pudding, custard, pop tarts, granola/protein bars | -0.10 |
| Whole grains^3^ | Whole grain cold cereals, oatmeal, hot-air popcorn, brown rice, whole wheat tortilla, whole wheat bread | 0.01 |
| Starchy vegetables | Casava, plantains (boiled or baked), corn, green peas, potatoes, winter squash, tannier, turnip, pasteles | 0.03 |
| Fast foods | Fast food hamburgers, chicken sandwiches, fried chicken, fish fillet sandwiches, French fries, onion rings, breakfast sandwiches, pizza, turnovers, condensed can soup | -0.07 |
| Fried foods | Fried rice, fish, beef, clams, crab, scallops, shrimp, crayfish, chicken, plantains, eggrolls, doughnuts, hashed browns | -0.07 |
| Non-saturated oil | Avocado, guacamole, green and black olives | 0.08 |
| Condiments | High salt: gravy (canned), horseradish, mustard, dill pickles, soy sauce | 0.08 |
| Eggs | Eggs: scrambled, fried, boiled, salad | 0.12 |
| Diet beverages | Diet soda, diet bottled or powdered iced tea, misc. diet drinks (Slim Fast) | -0.02 |
| Supplement score^4^ | Vitamins A, B-12, B-6, C, D, E; folate niacin, riboflavin, calcium, magnesium, selenium, thiamin, zinc, β carotene, iron | -0.03 |
| LIS Components^5^ | | |
| Heavy drinker | >1 drink for women; >2 drinks for men versus none | -0.26 |
| Moderate drinker | 1 drink for women; 1-2 drinks for men versus none | -0.42 |
| Physically active | Any activity versus sedentary | -0.63 |
| Current smoker | Current smoker versus does not currently smoke | 0.34 |
| Too little sleep | 5-6 hours of sleep versus 7-8 hours | 0.04 |
| Too much sleep | 9-10+ hours of sleep versus 7-8 hours | 0.05 |
| High stress | High perceived stress versus low | 0.03 |

^1^Weights are estimates obtained from multivariable linear regression models performed in the BPRHS cohort, representing the average change in an inflammation biomarker score (a summed score comprised of logged and z-score standardized hsCRP, IL-6, TNFα) per 1 SD increase in a dietary component or the presence of a lifestyle component. A positive estimate suggests that component has a proinflammatory effect while a negative estimate suggests an anti-inflammatory effect. The final regression model was adjusted for age, sex and estrogen status, blood glucose level (above and below 126 mg/dL), history of heart disease, history of cancer, white blood cell count, urinary cortisol, and all components of the DIS and LIS.

^2^Mean servings per day from baseline and wave 2 dietary intake were z-score standardized by sex

^3^ Defined as whole grain first ingredient; added sugar content was ≤ 12g/serving and DFIB was ≥ 3g/serving

^4^Supplement intakes were based on multivitamin and mineral use. Individuals were ranked into groups based on the Office of Dietary Supplements (ODS) recommended intake of each micronutrient.

^5^Lifestyle components were coded as categorical variables with “0” representing the referent category and “1” and/or “2” the nonreferent category.
