# Supplementary material for: Performance of cohort-adapted dietary and lifestyle inflammation scores among Hispanic adults
Source: Front Nutr. 2026 Jan 8;12:1675057. doi: 10.3389/fnut.2025.1675057 (PMC12823488; doi:10.3389/fnut.2025.1675057)
Supplement: Supplementary file 2 [file Table_2.DOCX]

**Supplementary Table 2.** Derived Dietary Inflammatory Score and Lifestyle Inflammatory Score component weights using data from the Boston Puerto Rican Health Study (2004-2012) including BMI as a covariate in the weight-building model.

| Component Group | Weights^1^ |
| --- | --- |
|  |  |
| Fruits and vegetables | 0.03 |
| 100% Fruit juice | 0.01 |
| Legumes | 0.06 |
| Fish, seafood, and starchy vegetables | 0.03 |
| Poultry | -0.13 |
| Red and organ meats | 0.10 |
| Processed foods | 0.05 |
| Added sugars, coffee and tea | 0.00 |
| High-fat dairy | -0.13 |
| Low-fat dairy and whole grains^2^ | 0.10 |
| Nuts and seeds | -0.08 |
| Non-saturated oil | 0.10 |
| Eggs | 0.08 |
| Condiments | 0.08 |
| Diet beverages | -0.07 |
| Supplement score^3^ | -0.01 |
| Heavy drinker | 0.01 |
| Moderate drinker | -0.51 |
| Physically active | -0.55 |
| Current smoker | 0.67 |
| Too little sleep | -0.04 |
| Too much sleep | -0.07 |
| High stress | 0.05 |

^1^Weights are estimates obtained from multivariable linear regression models performed in the BPRHS cohort, representing the average change in an inflammation biomarker score (a summed score comprised of logged and z-score standardized hsCRP, IL-6, TNFα) per 1 SD increase in a dietary component or the presence of a lifestyle component. A positive estimate suggests that component has a proinflammatory effect while a negative estimate suggests an anti-inflammatory effect. The final regression model was adjusted for age, sex and estrogen status, BMI, blood glucose level (above and below 126 mg/dL), history of heart disease, history of cancer, white blood cell count, urinary cortisol, and all components of the DIS and LIS.

^2^ Defined as whole grain first ingredient; added sugar content was ≤ 12g/serving and DFIB was ≥ 3g/serving

^3^ Supplement intakes were based on multivitamin and mineral use. Individuals were ranked into groups based on the Office of Dietary Supplements (ODS) recommended intake of each micronutrient.
