# Supplementary material for: Performance of cohort-adapted dietary and lifestyle inflammation scores among Hispanic adults
Source: Front Nutr. 2026 Jan 8;12:1675057. doi: 10.3389/fnut.2025.1675057 (PMC12823488; doi:10.3389/fnut.2025.1675057)
Supplement: Supplementary file 3 [file Table_3.DOCX]

**Supplementary Table 3.** Comparison of factor loading distance matrix mean and SD for 3 factors from BPRHS (2004-2012).
