# Supplementary material for: Performance of cohort-adapted dietary and lifestyle inflammation scores among Hispanic adults
Source: Front Nutr. 2026 Jan 8;12:1675057. doi: 10.3389/fnut.2025.1675057 (PMC12823488; doi:10.3389/fnut.2025.1675057)
Supplement: Supplementary file 4 [file Table_4.DOCX]

**Supplementary Table 4.** Associations between dietary and lifestyle inflammation scores and high inflammation (high sensitivity C-reactive protein) in the PROSPECT cohort, including BMI as a covariate in the weight-building model

| Inflammation Scores | Outcome Variable Dichotomized^1^ | Outcome Variable Continuous^2^ |
| --- | --- | --- |
|  | Adjusted OR (95% CI) | β (95% CI) |
| DIS^3^ | 0.97 (0.58, 1.6) | 0.02 (-1.99, 0.23) |
| LIS^4^ | 1.35 (0.93, 2.0) | 0.16 (0.00, 0.31) |

^1^Serum hsCRP concentration dichotomized at ≤/> 3mg/L

^2^Serum hsCRP log transformed

^3^models adjusted for the following covariates: age, sex and estradiol status,

perceived stress, sleep, smoker status, alcohol intake, physical activity,

total energy, history of diabetes, heart disease, cancer, education

^4^models adjusted for the following covariates: age, sex and estradiol status,

history of diabetes, heart disease, cancer, education

Box indicates p-value less than 0.05
