# Supplementary material for: Performance of cohort-adapted dietary and lifestyle inflammation scores among Hispanic adults
Source: Front Nutr. 2026 Jan 8;12:1675057. doi: 10.3389/fnut.2025.1675057 (PMC12823488; doi:10.3389/fnut.2025.1675057)
Supplement: Supplementary file 5 [file Table_5.DOCX]

**Supplementary Table 5.** Factor loadings for dietary patterns from the exploratory factor analysis BPRHS (2004-2012).

| Factor 1 | |  | Factor 2 | |  | Factor 3 | |
| --- | --- | --- | --- | --- | --- | --- | --- |
| Fruits, vegetables, low-fat dairy, whole grains  (Healthy) | |  | Saturated fat, vegetables, poultry, tomatoes, legumes, fish (Traditional) | |  | Processed and red meat, fast and fried food, eggs, processed dairy (Industrialized) | |
| Food group^1^ | Factor loadings |  | Food group^1^ | Factor loadings |  | Food group^1^ | Factor loadings |
|  |  |  |  |  |  |  |  |
| Yellow & orange fruits & vegetables | 0.62 |  | Saturated fat | 0.73 |  | Processed meat | 0.43 |
| Other fruit | 0.60 |  | Other vegetables | 0.72 |  | Fast foods | 0.40 |
| Apples & berries | 0.55 |  | Poultry | 0.40 |  | Fried foods | 0.39 |
| Green vegetables | 0.55 |  | Tomatoes | 0.35 |  | Eggs | 0.33 |
| Low-fat dairy | 0.43 |  | Greens | 0.28 |  | Red meat | 0.30 |
| Whole grains | 0.42 |  | Legumes | 0.25 |  | Processed dairy | 0.28 |
| Processed meat | -0.20 |  | Fish | 0.22 |  | High-fat dairy | -0.20 |
| Legumes | -0.22 |  | Added sugar | -0.37 |  | Saturated fat | -0.20 |
| Red meat | -0.31 |  | High-fat dairy | -0.39 |  | Other fruit | -0.22 |
| Refined grains | -0.34 |  |  |  |  | Legumes | -0.31 |

^1^ Components of food groups provided in Table 3
