# Supplementary material for: Performance of cohort-adapted dietary and lifestyle inflammation scores among Hispanic adults
Source: Front Nutr. 2026 Jan 8;12:1675057. doi: 10.3389/fnut.2025.1675057 (PMC12823488; doi:10.3389/fnut.2025.1675057)
Supplement: Supplementary file 6 [file Table_6.DOCX]

**Supplementary Table 6.** Factor loadings for dietary patterns from the confirmatory factor analysis PROSPECT (2019-2021).

| Factor 1 | | Factor 2 | | Factor 3 | |
| --- | --- | --- | --- | --- | --- |
| Fruits, vegetables, low-fat dairy, whole grains (Healthy pattern) | | Saturated fat, vegetables, poultry, tomatoes legumes, fish (Traditional pattern) | | Processed and red meat, fast and fried foods, eggs, processed dairy (Industrialized pattern) | |
| Food group | Factor loadings | Food group | Factor loadings | Food group | Factor loadings |
| Yellow & orange fruits & vegetables | 0.76 | Red meat | 0.77 | Red meat | 0.76 |
| Green vegetables | 0.57 | Other vegetables | 0.71 | Fast food | 0.36 |
| Other fruit | 0.49 | Other fat | 0.71 | Processed meat | 0.35 |
| Apples & berries | 0.43 | Tomatoes | 0.27 | Fried food | 0.27 |
| Nuts & seeds | 0.40 | Added sugar | -0.36 | Processed dairy | 0.27 |
| Whole grains | 0.27 | High-fat dairy | -0.45 | High-fat dairy | -0.35 |
| Low-fat dairy | 0.22 |  |  | Legumes | -0.45 |
| Refined grains | -0.20 |  |  |  |  |
| Other fat | -0.27 |  |  |  |  |
