# Supplementary material for: Performance of cohort-adapted dietary and lifestyle inflammation scores among Hispanic adults
Source: Front Nutr. 2026 Jan 8;12:1675057. doi: 10.3389/fnut.2025.1675057 (PMC12823488; doi:10.3389/fnut.2025.1675057)
Supplement: Supplementary file 7 [file Table_7.DOCX]

Supplementary Table 7. Associations between dietary factors 1-3 and high inflammation (biomarker score or high sensitivity C-reactive protein) in the BPRHS (wave 2) and PROSPECT cohorts

| Factor Quintiles | Outcome Variable^1^ Dichotomized  BPRHS | Outcome Variable^1^ Continuous  BPRHS | Outcome Variable Dichotomized^2^  PROSPECT | Outcome Variable Continuous^2^  PROSPECT |
| --- | --- | --- | --- | --- |
|  | Adjusted OR^3^ (95% CI) | β (95% CI)^3^ | Adjusted OR^4^ (95% CI) | β (95% CI)^4^ |
| Factor 1  (Healthy) |  |  |  |  |
| Q1 (ref) |  |  |  |  |
| Q2 | 0.73 (0.45, 1.2) | -0.08 (-0.52, 0.36) | 0.79 (0.47, 1.3) | -0.13 (-0.30, 0.05) |
| Q3 | 0.92 (0.58, 1.5) | 0.11(-0.33, 0.55) | 0.90 (0.53, 1.5) | -0.12 (-0.29, 0.06) |
| Q4 | 0.89 (0.55, 1.4) | -0.12 (-0.57, 0.33) | 1.0 (0.61, 1.7) | -0.03 (-0.21, 0.14) |
| Q5 | 0.60 (0.37, 0.97) | -0.41 (-0.86, 0.05) | 0.74 (0.44, 1.3) | -0.12 (-0.30, 0.05) |
| Factor 2  (Traditional) |  |  |  |  |
| Q1 (ref) |  |  |  |  |
| Q2 | 0.73 (0.46, 1.2) | -0.12 (-0.56, 0.33) | 0.88 (0.52, 1.5) | -0.09 (-0.27, 0.09) |
| Q3 | 0.93 (0.59, 1.5) | -0.17 (-0.60, 0.26) | 1.2 (0.70, 2.0) | -0.06 (-0.24, 0.11) |
| Q4 | 0.88 (0.55, 1.4) | 0.01 (-0.43, 0.44) | 0.89 (0.52, 1.5) | -0.13 (-0.32, 0.05) |
| Q5 | 1.3 (0.80, 2.0) | 0.26 (-0.18, 0.71) | 0.76 (0.44, 1.3) | -0.15 (-0.33, 0.03) |
| Factor 3  (Industrialized) |  |  |  |  |
| Q1 (ref) |  |  |  |  |
| Q2 | 0.81 (0.51, 1.3) | -0.11 (-0.54, 0.32) | 0.90 (0.53, 1.5) | 0.05 (-0.12, 0.23) |
| Q3 | 1.2 (0.78, 1.9) | 0.41 (-0.02, 0.84) | 1.4 (0.81, 2.3) | 0.12 (-0.06, 0.3) |
| Q4 | 1.20 (0.75, 1.9) | 0.30 (-0.14, 0.75) | 0.70 (0.41, 1.2) | -0.06 (-0.24, 0.12) |
| Q5 | 1.0 (0.63, 1.7) | -0.05 (-0.52, 0.42) | 1.1 (0.61, 1.8) | -0.02 (-0.20, 0.17) |

^1^Inflammation biomarker score dichotomized at ≤/> -0.23 for OR; continuous for β coefficients

^2^Serum hsCRP concentration dichotomized at ≤/> 3mg/L for OR; log transformed & continuous for β

^3^models adjusted for the following covariates: age, sex and estradiol status, BMI, smoker status,

physical activity, history of diabetes and heart disease

^4^models adjusted for the following covariates: age, sex and estradiol status, BMI, smoker status,

history of diabetes, perceived stress

Box indicates p-value less than 0.05
