# Supplementary material for: Performance of cohort-adapted dietary and lifestyle inflammation scores among Hispanic adults
Source: Front Nutr. 2026 Jan 8;12:1675057. doi: 10.3389/fnut.2025.1675057 (PMC12823488; doi:10.3389/fnut.2025.1675057)
Supplement: Supplementary file 8 [file Data_Sheet_1.docx]

**Supplementary Methods**

The mean value of hsCRP measured from blood collected at wave 2 of the BPRHS, and from the BPROS (approximately 1-2 years after the blood draw at wave 2). If a participant had hsCRP data from only one timepoint, that value was used as an estimate of mean intake (*n* = 330). hsCRP was assessed from serum using the Immulite 1000 High Sensitivity CRP kit (LKCRP) on the Immulite 1000 machine (Siemens Medical Solutions Diagnostics, Los Angeles, CA. The intra- and inter assay CVs (%) were reported as 5.2% and 7.3% (31). Plasma TNFα and IL-6 were measured by ELISA using the Quantikine ELISA kit, R&D Systems, Minneapolis, MN. Reported intra- and inter assay CVs (%) for TNFα were 5.3% and 10.8% and for IL-6 they were 1.6% and 3.3% (31). Data were checked for implausible laboratory reference ranges and/or acute infection that could falsely indicate inflammation. Implausibly high values were identified as outliers and removed from the analysis (1 hsCRP value at 240.8 mg/L and 1 TNFα value at 78.4 pg/mL). Acute infection in participants with hsCRP > 40mg/L was checked by a comparison with white blood cell (WBC) count (cohort range: 3,300 to 9,000 cells/mL; normal range: 4,500 to 11,000 cells/mL (32);) therefore, there were no outliers in the inflammatory data identified due to infection.

WBC count was measured from blood collected at the wave 2 visit of the BPRHS using electronic impedance, light scatter, Double Hydrodynamic Sleeving System on the HORIBA ABX Pentra 60 C+ (ABX Diagnostics, Irvine, California 92618). Glucose was measured from blood collected at the wave 2 BPRHS visit using an enzymatic, kinetic reaction on the Olympus AU400e with Olympus Glucose Reagents (OSCR6121) (Olympus America Inc., Melville, NY) (22). Cortisol was measured in urine samples from the BPRHS wave 2 visit. A 12-hour urine sample was collected the morning after the home interview, with the 12-hour period beginning in the evening and ending the next morning. Cortisol analysis was made using an immunoenzymatic colorimetric method with an ALPCO cortisol assay (ALPCO, 26-G Keewaydin Dr, Salem, NH 03079) and was corrected for urine volume and creatinine excretion with the following equation: (cortisol*urine volume/creatinine excretion)/2.3.

In the PROSPECT cohort, hsCRP was measured using an immunoturbidimetric method (51 Calle Palma Arecibo, Puerto Rico) and was not collected from 310 participants. All but two outlier hsCRP values were retained (151.2 and 151.9) based on the plausibility of the values in this cohort. Dietary data and blood were collected at the same visit.
